# Transcriptomic Profile of Canine Mammary Ductal Carcinoma

**DOI:** 10.3390/ijms24065212

**Published:** 2023-03-08

**Authors:** Driéle B. Santos, Geysson J. Fernandez, Luciana M. C. Pardini, Maria Inês M. C. Pardini, Adriana C. Ferrasi

**Affiliations:** 1 Department of Internal Medicine, Botucatu Medical School (FMB), São Paulo State University (UNESP), Botucatu 01049-010, SP, Brazil; 2 Grupo Biologia y Control de Enfermedades Infeciosas (BCEI), Facultad de Ciencias exactas y Naturales, Universidad de Antioquia (UdeA), Medellín 050010, Colombia; 3 Hospital Veterinário de São Bento, 1200-822 Lisboa, Portugal

**Keywords:** mammary ductal carcinoma, transcriptome, female dogs

## Abstract

Dogs can be excellent models for spontaneous studies about breast cancers, presenting similarities in clinical behavior and molecular pathways of the disease. Thus, analyses of the canine transcriptome can identify deregulated genes and pathways, contributing to the identification of biomarkers and new therapeutic targets, benefiting humans and animals. In this context, this study aimed to determine the transcriptional profile of canine mammary ductal carcinoma and contribute to the clarification of the importance of deregulated molecules in the molecular pathways involved in the disease. Therefore, we used mammary ductal carcinoma tissue samples and non-tumor mammary tissue from the radical mastectomy of six female dogs. Sequencing was performed on the NextSeq-500 System platform. A comparison of carcinoma tissue and normal tissue revealed 633 downregulated and 573 upregulated genes, which were able to differentiate the groups by principal component analysis. Gene ontology analysis indicated that inflammatory, cell differentiation and adhesion, and extracellular matrix maintenance pathways were mainly deregulated in this series. The main differentially expressed genes observed in this research can indicate greater disease aggressiveness and worse prognosis. Finally, the study of the canine transcriptome indicates that it is an excellent model to generate information relevant to oncology in both species.

## 1. Introduction

Cancer is the leading cause of death in humans and their domestic companions [1,2]. Breast carcinoma was the second most incident cancer (2.2 million new cases), causing 685,000 human deaths in 2020 [2]. Something similar can be observed among companion animals, especially dogs, since most tumors in uncastrated female dogs are mammary [3,4], with 50 to 75% being malignant [4].

Canine and human mammary cancers share several biological characteristics, such as histology, pathophysiology, clinical behavior, and molecular pathways [5,6]. Genomic analyses in canine tumors have revealed similarities between the two species, providing new insight into the genetic basis of such tumors [7]. Interestingly, several genes linked to increased risk of human cancers have also been observed in canines, such as mutations in BRCA1/2 [8,9,10] that predispose to breast and ovarian cancer in humans. The scenario presented above, combined with the shared home environment, makes modern dogs a suitable model for comparative oncogenetic studies.

Despite the relevance of the disease and the available studies, there are still gaps to be filled with respect to the molecular mechanisms involved in canine mammary carcinoma. Thus, global transcriptomic research based on next-generation sequencing may contribute to a better understanding of the origins and progression of tumors, besides contributing to the identification of biomarkers with potential use in the diagnosis and prognosis and even as new therapeutic targets in cancer, benefiting humans and animals.

In this context, the aim of the present study was to determine the transcriptional profile of canine mammary ductal carcinoma samples based on next-generation sequencing and contribute to the clarification of the importance of deregulated molecules in the molecular pathways involved in the disease.

## 2. Results

We used mammary ductal carcinoma tissue samples and non-tumor mammary tissue from the radical mastectomy of six female dogs to identify deregulated genes and pathways with RNA-seq. Sequencing was performed on the NextSeq-500 System platform with four sequencing libraries and two tumoral libraries (T1 and T2) from tissues histologically classified as mammary ductal carcinoma [11] and two control libraries (C1 and C2) from adjacent non-tumor tissue. All libraries were composed of a pool of samples from three patients with early-stage disease (T1N0M0). Next, we performed gene ontology enrichment [12], identification of transcription factors [13], and in silico identification of the secretome [14].

The mean age of the animals in the first group (C1 and T1) was 10 years, and in the second group (C2 and T2) the mean age was 7.7 years, with an overall mean of 8.9 years. This result agrees with epidemiological studies, which indicate that age is one of the main risk factors for the development of breast cancer [3]. After 8 years of age, this risk starts to be significant and increases with age [15].

**Figure 1:**
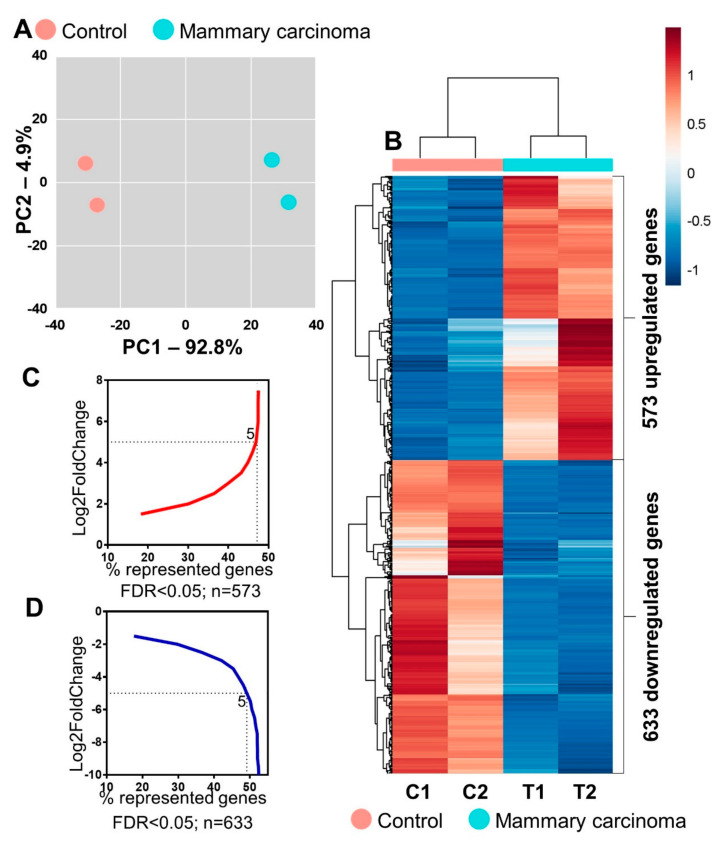
Transcriptional analysis of mammary carcinoma samples from female dogs showed a differential gene expression profile. (**A**) Principal component analysis of the expression of the control (pink) and mammary carcinoma (green) genes showed a high percentage variance between the control and breast carcinoma groups (PC1 = 92.8%) but low variance within each group (PC2 = 4.9%). (**B**) Heatmap with the 1206 differentially expressed genes between the two groups: control (pink) and mammary carcinoma (green). (**C**) Graph of log2fold change distribution of upregulated (red) and (**D**) downregulated (blue) genes.

Through global transcriptome analysis, we identified 11,278 genes among the 36,930 genes in the canine reference genome (RefSeq) [16]. Comparing mammary carcinoma and non-tumoral tissue samples, 1206 differentially expressed genes (DEgenes) (Supplementary Table S1) were found with statistical significance (*p* < 0.05 and −1 ≥ log2fold change ≤1), of which 633 were negatively regulated (downregulated) and 573 were positively regulated (upregulated) (Figure 1A–D). This transcriptional profile can distinguish each group by principal component analysis (PCA) (Figure 1A), as in the heatmap shown in Figure 1B.

**Figure 2:**
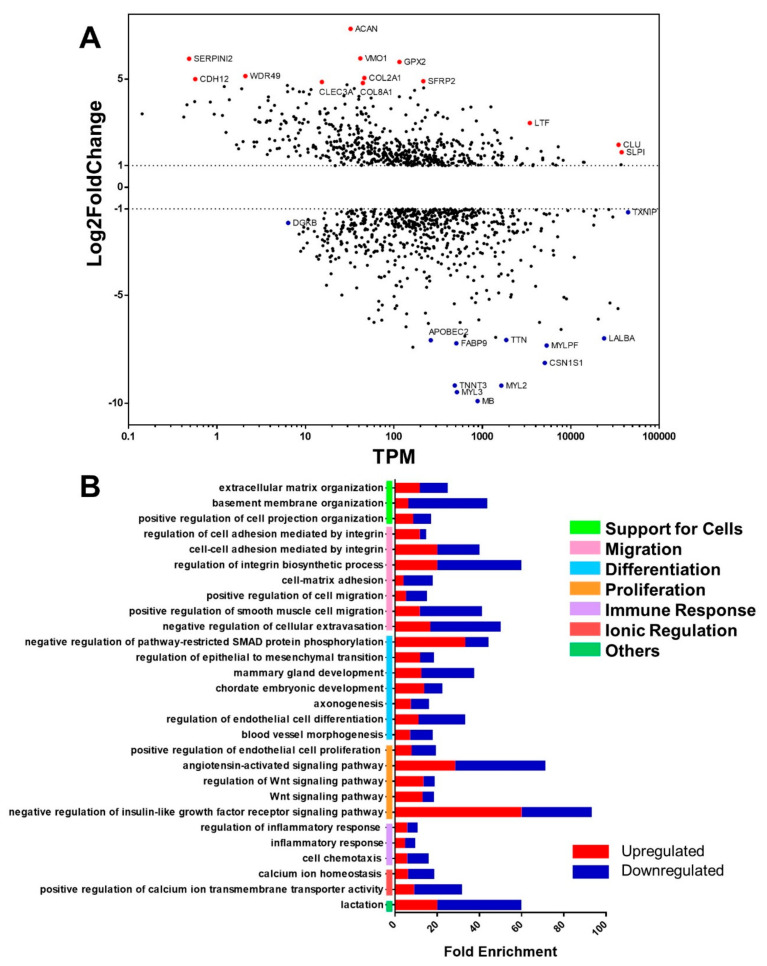
Genes and cell pathways associated with mammary carcinoma in female dogs. (**A**) MA scatter plot that indicates the mean count abundance (TPM, transcripts per million) and the degree of regulation (log2fold change). Genes with a log2fold change >1 are overexpressed, and genes with a log2fold change <1 are underexpressed in the mammary carcinoma group compared to normal tissue. The genes that showed greater abundance and a higher degree of positive regulation are highlighted in red, and the abundant genes with a high degree of negative regulation are highlighted in blue. (**B**) Analysis of differentially expressed gene enrichment in breast carcinoma, highlighting the main affected pathways. The colored bars indicate the percentage of upregulated (red) and downregulated (blue) genes among the total genes of each pathway.

From the MA plot, which comprises the expression change level (log2fold change) on the y-axis and the mean abundance (TPM—transcripts per million) on the x-axis (Figure 2A), it was possible to identify abundant genes and genes with a high degree of deregulation (positive and negative). The most abundant genes showed lower levels of expression change, such as the upregulated gene CLU, which encodes a chaperone; SLPI, which encodes a protease inhibitor; and the downregulated TXNIP, a tumor suppressor that encodes a protein that protects the cell from oxidative stress. Most abundant genes (TPM ≥ 1000) with a high degree of expression change (−5 ≥ log2fold change ≥5) are downregulated. The most upregulated genes, on the other hand, are less abundant (TPM < 100), such as SERPINI2, which encodes a protease inhibitor protein; CDH12, which, despite its role in cell adhesion and proliferation, is not commonly studied in breast cancer; and WDR [16], a gene about which little is known. This MA plot profile is typical of cancers since the cell undergoes major changes, so highly upregulated genes are not common in that cell type, resulting in highly expressed genes that are not very abundant and vice-versa, as shown in Figure 2A.

In the MA, we highlighted the 10 most and 10 least expressed genes among those differentially expressed in the comparative analysis between the mammary carcinoma and control groups; we observe that the downregulated genes had a log2fold change <−7, in contrast to the upregulated genes, which had a log2fold change >5. Most of the upregulated genes are related to tissue organization by encoding extracellular matrix component proteins (ACAN, VMO1, COL2A1, and COL8A1), as well as cell–cell (CDH12) and cell–extracellular matrix (CLEC3A) adhesion proteins. There are also genes involved in protecting the cell against oxidative stress (SERPINI2 and GPX2) and cell differentiation via the Wnt signaling pathway (SFRP2).

Most of those downregulated genes participate in muscle contraction by encoding skeletal and cardiac muscle components (TTN, MYLPF, TNNT3, MYL2, and MYL3) and the protein responsible for oxygen transport in these tissues (MB). In addition, some other downregulated genes are associated with the expression of genes involved in lipid metabolism (FABD9), encoding the major milk protein (CSN1S1) and an enzyme participating in mRNA editing (APOBEC2).

In the gene ontology (Figure 2B), 28 relevant pathways were identified, which participate in tissue maintenance and the cell cycle and can be subdivided between cell support, migration, differentiation, proliferation, immune response, and ion regulation groups. In each pathway of the ontology, we analyzed the percentage of up- and downregulated genes differentiated by color, where red represents the percentage of upregulated genes and blue represents the percentage of downregulated genes. Thus, we observed that the cell differentiation pathways have the most downregulated genes, while the embryonic development pathway has the most upregulated genes. Furthermore, the most enriched pathways are related to cell adhesion, regulation of growth factor signaling, angiotensin signaling, and lactation.

The cell chemotaxis pathway was included in the immune response set because it represents other pathways inducing positive chemotaxis of leukocytes, monocytes, and mast cells, which participate in the inflammatory response.

**Figure 3:**
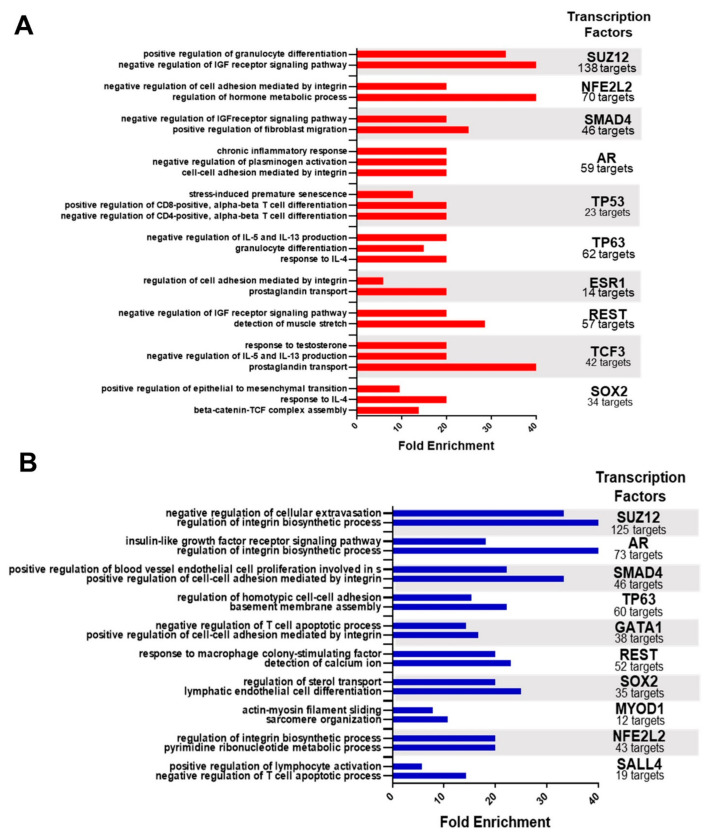
Regulation pathways of differentially expressed transcription factors. (**A**) Enrichment analysis of target genes of the transcription factors that upregulate genes in mammary carcinoma. (**B**) Enrichment analysis of target genes of the transcription factors that downregulate genes in breast carcinoma. The main affected pathways of the main transcription factors and the number of targets are highlighted. The colored bars indicate the percentage of upregulated (red) and downregulated (blue) genes among the total genes of each pathway; the transcription factors that regulate the number of targets associated with the enriched pathways are highlighted on the right.

From the query of DEgenes in X2K Web, the regulation networks for the up- and downregulated genes were defined separately. In this way, 98 transcription factors were found that regulate the set of upregulated genes and 95 that regulate the downregulated genes (Supplementary Tables S2 and S3). Next, the ten transcription factors that most regulate each set (up- and downregulated genes) and their targets were highlighted, and the ontology of these targets was determined (Figure 3). The ontology of the upregulated targets (Figure 3A) indicated mainly immune, tissue organization, and hormonal pathways, highlighting the negative regulation of IL-5 and IL-13 production, the response to IL-4, the response to chronic inflammation, and the differentiation of immune system cells (granulocytes and TCD4 and TCD8 lymphocytes). Tissue organization comprises the pathways of negative regulation of plasminogen activation, positive regulation of fibroblast migration, integrin-mediated regulation and cell adhesion, assembly of the β-catenin-TCF complex, and regulation of the epithelial–mesenchymal transition (EMT). The hormonal pathways are the most enriched, among which we observed the regulation of the metabolic process of hormones, prostaglandin transport, and cellular response to testosterone stimulation, in addition to the regulation of the IGF receptor signaling pathway.

In the ontology of downregulated targets (Figure 3B), we mainly observed pathways of tissue organization and cell adhesion, processes that participate in the cell cycle, immune pathways, and muscle cell activity. The regulation of the integrin biosynthesis process and integrin-mediated cell adhesion are the most enriched pathways and those most associated with the transcription factors that regulate DE genes. It is also important to consider the formation of the basement membrane, the negative regulation of cell leakage, and endothelial cell proliferation in the budding process of angiogenesis among the pathways of tissue organization. The cell cycle processes range from the pyrimidine metabolic process and calcium ion sensing to the IGF receptor signaling pathway. The immune pathways include negative regulation of T-cell apoptosis, positive regulation of lymphocyte activation, and response to macrophage colony-stimulating factor. The pathways involved in muscle cell activity (actin–myosin filament sliding and sarcomere organization) are specific to the transcription factor MYOD1.

Interestingly, most of the highlighted transcription factors regulate both upregulated and downregulated genes, such as SUZ12, NFE2L2, SMAD4, TP63, REST, and AR (which is part of the DESeq set). These transcription factors regulate the same functions between the sets (up- and downregulated genes), although through different targets and pathways.

**Figure 4:**
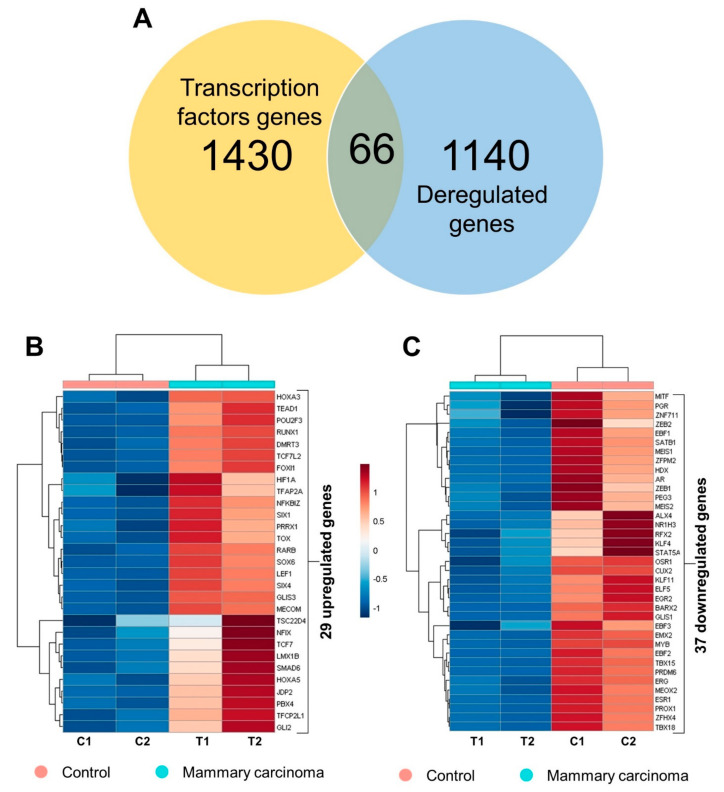
Transcriptional analysis of mammary carcinoma samples from female dogs showed differentially expressed transcription factor genes. (**A**) Venn diagram discriminating the differentially expressed genes found in the performed analyses and all the genes that encode transcription factors according to the Human Protein Atlas database. (**B**) Heatmap with the 29 genes of upregulated transcription factors in canine mammary carcinoma; control (in pink) and mammary carcinoma (in green). (**C**) Heatmap with the 37 transcription factor genes. Analysis of target genes of the five main transcription factors within the set that regulate upregulated genes and downregulated genes.

Among the differentially expressed genes, 66 transcription factors (Figure 3) were found (Figure 4A), of which 29 are upregulated (Figure 4B), with two identified as regulators of the total set, and 37 are downregulated (Figure 4C), with six being regulators of the DEgenes set, which is the case of the AR gene.

**Figure 5:**
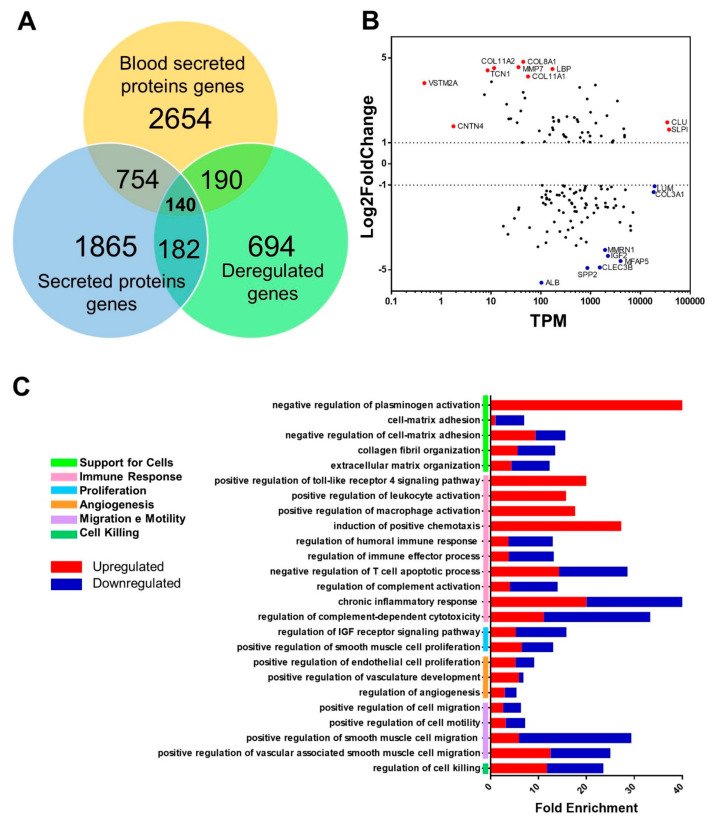
Highlighting genes encoding secreted proteins and cellular pathways associated with mammary carcinoma in female dogs as potential biomarkers. (**A**) Venn diagram that discriminates the differentially expressed genes found in the performed analyses, all the genes that encode secreted proteins, and all the genes that encode proteins specifically secreted in the blood according to the Human Protein Atlas database. (**B**) MA plot of the 140 genes that encode proteins secreted in the blood, with those genes with a high degree of positive regulation highlighted in red and those genes with a high degree of negative regulation highlighted in blue. (**C**) Analysis of the enrichment of genes encoding secreted blood proteins found to be differentially expressed in breast carcinoma, highlighting the main affected pathways. The colored bars indicate the percentage of upregulated (red) and downregulated (blue) genes among the total genes of each pathway.

Based on the data from the Human Protein Atlas, a Venn diagram was drawn with the set of DEgenes, the total secreted protein gene, and blood-secreted protein gene data (Figure 5A); thus, we identified 140 possible circulating protein genes that could be used as biomarkers. Among these genes, the ten most and least expressed genes in the MA graph were highlighted (Figure 5B).

Among this set of genes that encode proteins secreted in the blood, the most expressed genes encode collagen (COL11A2 and COL8A1); however, the MMP7 also stands out, which encodes a metalloproteinase responsible for the breakdown of the extracellular matrix for tissue remodeling, as well as the LBP gene, which encodes a protein that binds to Gram-negative bacteria wall lipopolysaccharides and regulates the immune response. In addition, some genes are not very abundant (TPM < 10) but have high expressions, such as VSTM2A, which encodes a protein whose function is to regulate preadipocyte differentiation, and CNTN4, which encodes an axon-associated adhesion molecule.

On the other hand, the least expressed gene is ALB, which encodes albumin, and is already associated with a worse prognosis in women with breast cancer [17]. Also noteworthy are the genes SPP2, which encodes a protein of the cystatin superfamily, and CLEC3B, which encodes a lectin type C member 3 of family 3, which is associated with metastasis and tumor invasion [18] in other cancers, such as hepatocellular carcinoma. Moreover, there are very abundant genes (TPM > 10,000) that have low expression, as is the case of genes LUM, which encodes the lumican protein, which regulates the organization of collagen fibrils, and COL3A1, which encodes the α1 chain of collagen type 3 and is found mainly in connective tissues of organs such as the lungs, uterus, and vascular system.

Gene ontology of the set of 140 genes encoding proteins secreted in the blood was performed (Figure 5C). In this analysis, 26 relevant pathways were identified, which are associated with immune response, extracellular matrix organization, angiogenesis, and cell cycle processes such as proliferation, migration, and cell death. We observed that the signaling and activation pathways of immune system cells and chemotaxis of these cells are enriched with only upregulated genes. The negative regulation pathway of plasminogen activation also comprises only upregulated genes and is one of the most enriched pathways. Other more enriched pathways are the pathways of response to chronic inflammation and regulation of cytotoxicity of the complement system. However, the angiogenesis-related pathways were the least enriched, while the set of extracellular matrix organization pathways is well-enriched and common to all performed ontologies. It is possible to separate them into pathways related to cell adhesion, the organization of extracellular matrix components, and their degradation, which allows for cell invasion.

## 3. Discussion

With RNA-seq, we obtained four sequencing libraries, two tumoral libraries (T1 and T2) from tissues histologically classified as mammary ductal carcinoma [11], and two control libraries (C1 and C2) from adjacent non-tumor tissue. When comparing carcinoma tissue and normal tissue, we found 633 downregulated and 573 upregulated genes, which were able to differentiate the groups by PCA.

These results indicate that some of the genes highlighted in the MA plot can be related to a poor prognosis in human breast cancer and other carcinomas, mainly by stimulating invasion capacity and metastasis at a distance and to lymph nodes. This are the case with the high expression of CLU and SLPI, upregulated genes in the tumor samples (Figure 2A) that are correlated to cell migration and invasion capacity, favoring metastasis and therefore resulting in more severe disease [19,20,21]. CLU encodes the protein isoform sCLU, exhibits chaperone activity, so it is overexpressed in stress situations to ensure the protection of cells from apoptosis through p53-dependent and p53-independent pathways. In addition, it participates in extracellular matrix organization and contributes to the ease of cells’ intravasation of blood and/or lymphatic vessels because it modulates the activity of MMP9 [22]. On the other hand, SLPI can prevent tissue destruction and regulates the inflammatory response [23]. This protein can also induce vasculogenic mimicry [21], a type of angiogenesis characterized by the formation of microvascular channels composed of tumor cells, which feed the tumor and allow for the perfusion of cells through the vessels [21,24].

Another interesting transcript is SERPINI2, which is overexpressed, although not very abundant (Figure 2A). This gene encodes a myoepithelial serine proteinase inhibitor (MEPI), which is indirectly involved in reducing plasminogen activation, whose negative regulation pathway is highly enriched. This plasminogen protection occurs in cells with high expression of SERPINI2, as observed in our results. Studies indicate that in breast tumors, the expression of this gene is not observed; however, with transfection of MEPI cDNA into breast neoplastic cells, inhibition of invasion is observed [25].

These results also draw attention to the WDR49 transcript, which is encoded by a gene that is not commonly studied in breast cancer and with scarce information available about its specific functions [26]. Furthermore, it participates in a family of genes that encode proteins regulating various cellular functions, such as cell division and cell fate determination, ontology-enriched pathways, transcription, transmembrane signaling, mRNA modification, and vesicle fusion [27,28]. However, in a GWAS study of patients with hepatocellular carcinoma and chronic hepatitis B virus (HBV) infection, Han et al. (2020) reported low expression of WDR49 [29].

The proteolysis of the extracellular matrix, represented, for example, by the high expression of MMP7 (Figure 5B), is relevant for cell invasion and metastasis. However, excessive connective tissue formation is characteristic of human mammary ductal carcinoma [30], and the density and organization of collagen fibrils can regulate the progression of mammary tumors in humans, mice, and dogs [31]. We observed high expression of several collagen types (COL2A1, COL8A1, COL11A1, and COL11A2). These collagens can be used as myoepithelial markers and are involved in the progression of canine mammary ductal carcinoma in situ to invasive carcinoma, mainly by activating the TGFβ signaling pathway, which is related to epithelial–mesenchymal transition (EMT), invasion, and stem cell characteristics [32,33,34]. Likewise, other overexpressed genes in breast carcinoma samples related to EMT are SFRP2 [32,33,34], which modulates Wnt signaling, a pathway highly enriched in ontologies that act on EMT, among other tumor development processes [34]; and CDH12 [35,36], which encodes a type 2 N-cadherin from the family of proteins responsible for calcium-mediated cell adhesion. This function is associated with gene ontology, since the cell adhesion, homeostasis, and calcium transport pathways are highly enriched.

Transcription factors are also involved in EMT, such as the high expression of ZEB1 related to the high expression of CDH12 [35]. ZEB1 regulates upregulated genes in breast tumor tissue; however, this transcription factor was found to be downregulated in the present study (Figure 4C). MECOM is one of the more upregulated genes encoding transcription factors. This transcription factor acts as a histone methyltransferase involved in the regulation of several cellular functions, including cell proliferation [37], as it is classified as a stem cell transcription factor by regulating a set of cadherins [29]. Thus, high expression of MECOM is related to an increased risk of early death. Moreover, the higher the expression, the more advanced the stage of the disease [38].

ACAN is also involved in extracellular matrix remodeling and showed the highest fold change in the mammary ductal carcinoma samples. It encodes a proteoglycan specific to cartilaginous tissue, as does COL2A1, as discussed earlier. There is evidence that the presence of these markers indicates metaplasia with cartilaginous characteristics [39,40]. In contrast, human mammary ductal carcinomas show no difference in ACAN expression [41], which is understandable, since metaplasia is uncommon in human breast cancers [39] but is frequent in benign tumors classified as mixed in dogs [11].

LUM encodes the lumican proteoglycan, a major component of the extracellular matrix, and plays an important role in angiogenesis, regulation of collagen fibrogenesis, cell invasion, and growth, which are important activities in tumor progression [33,40]. Its expression in breast cancer is controversial [40], but studies show that the downregulation of LUM in human breast cancers is associated with a worse prognosis [41]. Such an expression profile can be observed in the results reported herein, as despite being abundant, LUM was found to be downregulated.

Like LUM, TXNIP was abundant but downregulated in the analyzed tumor samples. This gene encodes the thioredoxin binding protein, which, by binding with thioredoxin, inhibits its function, causing the accumulation of reactive oxygen species, which are important for the control of oxidative stress in cells and, consequently, the induction of apoptosis and inhibition of cell proliferation [42,43]. Thus, it can be considered a tumor suppressor, and its reduced expression is associated with several cancers, such as hepatocellular carcinoma, lung cancer, and breast cancer in humans [43], mice, and dogs [44]. Knockdown of the TXNIP gene in MCF-7 cells causes a reduction in the inhibitory effect on cell growth [44].

The most downregulated genes are characteristic of muscle cells (MYL2, MYL3, MYLPF, TTN, and TNNT3). In a transcriptional study of tissues from different types of canine mammary carcinoma, muscle-related pathways such as cardiac muscle morphogenesis, skeletal muscle adaptation, and muscle adaptation were enriched and associated with downregulated genes in canine mammary ductal carcinoma tissues [45]. Similarly, several cell motility pathways, as well as extracellular matrix organization, were enriched and associated with upregulated genes [45]. Furthermore, these genes and proteins have been found to be significantly less expressed in breast cancers [45,46] and other carcinomas, such as oral [47,48] and tongue squamous cell cancer [49], as well as neck and head squamous cell cancer either in association with smoking or not [50,51].

On the other hand, high expression of TTN and MYL3 genes can be described as a biomarker of a worse prognosis in Ewing’s sarcoma [52]. TTN was also reported to be more expressed in gastric adenocarcinoma [53]. TTN gene and the protein encoded by it are large and therefore more susceptible to mutations and related to muscle weakness, heart disease, muscular dystrophy, and breast cancer [54]. Single-nucleotide polymorphisms (SNPs) in the TNN3 gene are associated with increased mammographic density, which is a risk factor for the development of breast carcinoma [55]. The abundant but most downregulated MB gene encodes the monomeric protein myoglobin, which is responsible for the transport and storage of O_2_ in muscle tissues. However, it has been shown that human breast epithelial cells also express this gene [56,57,58,59]. High expression of MB is observed in 40% of ductal carcinomas [58]; however, there are significant cases of downregulation of this gene in human breast cancer cells [59]. This indicates that MB may be a tumor suppressor associated with a better prognosis when upregulated because the higher the expression, the less aggressive the disease [58]. High expression of MB is associated with lower cell proliferation by affecting the dynamics of mitochondria, causing mitochondrial hyperfusion and preventing cell cycle progression from G1 to the S phase [59]. It also indicates a change in the energy matrix of the cell, since inactivation of the gene in vivo and in vitro leads to increased O_2_ uptake, energy substrate switch from fatty acids to glycolysis (which may be associated with the downregulated FABP9 gene) (therefore less O_2_ consumption), and increases in the activity of mitochondrial enzymes [56,58]. Although the molecular mechanisms related to MB are not yet well elucidated, this gene is commonly studied in breast and other hormone-dependent cancers such as prostate cancer because of its relationship with ERs (estrogen receptors). Just as long-lasting hypoxia leads to the overexpression of this gene, its downregulation seems to be related to estrogen exposure [57,58]. One study showed that when breast tumor cells expressing RE (RE+) were exposed to β-estradiol, they showed lower expression of MB, possibly via regulation of RUNX1 [56], one of the transcription factors observed to be differentially expressed in the present study and which regulates this set of genes.

The genes that encode proteins secreted into the blood and that were mostly downregulated and upregulated are ALB and LBP, respectively. Studies indicate that a decreased serum albumin level, a protein encoded by the ALB gene, is related to shorter survival in breast cancer patients [16,60,61], while high levels reduced the risk of death by 45% [62]. This effect may be due to albumin’s role in inhibiting estrogen-responsive tumor cell proliferation [63]. On the other hand, LBP encodes a lipopolysaccharide-binding protein, so it plays a role in the inflammatory response against bacteria by initiating the innate immune response. Therefore, it is related to inflammatory processes in the breast that lead to stromal remodeling and the development and progression of cancer in this tissue in humans [63] and mice [64]. Among these processes, breast involution [64], which resembles the wound healing process, and amyloidosis have been studied, with LBP showing amyloidogenic capacity [65].

We recognize the limitations of this study, including the need to validate the differential expression in a larger number of cases analyzed independently. Additionally, this study would be more informative if it had included samples of tumors in stages with different degrees of evolution. Such efforts are being pursued in our ongoing studies. Despite these limitations, our data indicate the main transcripts deregulated in canine mammary ductal carcinoma. As previously discussed, many of these genes have already been related to greater aggressiveness and worse prognosis in mammary carcinomas and other human and canine tumors, highlighting the ten most upregulated genes. Furthermore, such alterations allowed us to infer the main deregulated pathways, which will contribute to a better understanding of the disease in dogs. Finally, our study of the canine transcriptome seems to indicate that this is an excellent model to generate information relevant to oncology in both species.

## 4. Materials and Methods

### 4.1. Casuistry

Canine specimens were obtained from female dogs (*n* = 6) with naturally occurring primary mammary ductal carcinoma receiving treatment at the Veterinary Surgery and Anesthesiology Service (São Paulo State University (UNESP), Botucatu, SP, Brazil). Tumor tissue and adjacent non-tumoral tissue were obtained from surgeries that were part of standard treatment protocols. The histopathological classification as ductal mammary carcinoma (T1N0M0) was confirmed by two independent veterinary pathologists according to Goldschmidt et al. [11]. The female dogs included in this study were non-castrated, of different breeds (Poodle (1), Yorkshire Terrier (1), and mixed-breed (4)), and aged 5 to 14 years.

### 4.2. Ethical Premises

The study design and experimental protocols were approved by the Ethics Committee on the Use of Animals (CEUA) of the School of Veterinary Medicine and Animal Science of São Paulo State University (FMVZ-UNESP), Botucatu-SP, Brazil (protocol 49/2011) in accordance with the institutional policy and guidelines for ethical conduct in the care and use of animals.

All samples were obtained from surgeries that were part of standard treatment protocols following the attainment of written informed consent from the owners, and strict confidentiality of the owners and their dogs was maintained.

### 4.3. Methodology

Sequencing: The tissue was preserved in RNAlater at −70 °C until processing. First, 50 mg of tissue sample was triturated using a Precellys homogenizer^®®^ 24-Dual (Bertin Technologies, Rockville, Washington, DC, USA). We extracted the total RNA of the samples using TRIzol (Thermo Scientific, Waltham, MA, USA). Then, RNA was quantified using a Qubit RNA assay kit on a Qubit 2.0 fluorometer (Thermo Scientific, Waltham, MA, USA), and integrity (RIN > 8) was confirmed on an Agilent 2100 bioanalyzer (Agilent Technologies, Germany). Subsequently, this material was pooled, forming four groups: two tumoral groups (each composed of three samples of independent specimens) and two normal groups (each composed of three paired samples of normal tissue). We used each pool to build the four sequencing libraries. The cDNA libraries were built with a TruSeqTM Stranded Total RNA Sample Preparation Kit v2 (Illumina, San Diego, USA), and sequencing was performed on the NextSeq 500 System platform (Illumina, San Diego, USA) using a NextSeq 500/550 High Output v2 150 cycles (Illumina, San Diego, USA) sequencing kit according to the manufacturer’s instructions.

Statistical Analysis: First, the data obtained from sequencing were subjected to quality and cleanliness analysis. The minimum Phred score was 32 per position in the alignment. Then, we mapped the paired-end reads to the dog reference genome using TopHat2 with the following parameters: mate-inner-dist, 200; mate-std-dev, 100; no-novel-juncs; min-intron-length, 40. The number of counts was obtained using HTSeq with default settings and normalized by DESEq2 (version 1.26). Significantly differentially expressed genes were considered those with *p* < 0.05 and |log2fold change| ≥1. The abundance of mRNA transcripts was determined by converting transcripts per one million base pairs of mapped reads (TPM—transcripts per million).

Gene Ontology Enrichment (GO) was performed in the Enrichr platform (https://maayanlab.cloud/Enrichr/; accessed on 29 June 2021) [12] based on a hypergeometric test with Benjamini–Hochberg correction. We considered enriched processes with *p* < 0.05.

Identification of Transcription Factors: Using the X2K Web platform (https://maayanlab.cloud/X2K/; accessed on 21 August 2021) [13], it was possible to identify the upstream regulation networks of upregulated and downregulated genes separately, indicating the transcription factors that regulate such sets of genes. In addition, we distinguished which differentially expressed genes encoded transcription factors by comparing them to the Human Protein Atlas transcription factor database (www.proteinatlas.org; accessed on 21 August 2021) [14].

Identification of the Secretome: From the Human Protein Atlas database of secreted proteins, it was possible to identify which differentially expressed genes encode blood-secreted proteins, which could be potential non-invasive biomarkers.

## Data Availability

The datasets generated and/or analyzed during the current study are available in the National Center for Biotechnology Information repository at [https://www.ncbi.nlm.nih.gov/geo/query/acc.cgi?acc=GSE203589] (accessed on 10 January 2023) (accession number GSE203589). Additional information and tables are available as Supplementary Material.

## Supplementary Materials

The following supporting information can be downloaded at: https://www.mdpi.com/article/10.3390/ijms24065212/s1.
